# Pseudomelanoma: occult intraocular foreign body mimicking choroidal melanoma

**DOI:** 10.3205/oc000211

**Published:** 2023-01-30

**Authors:** Aslıhan Yılmaz Çebi, Bilge Batu Oto, Oğuzhan Kılıçarslan, Ahmet Murat Sarıcı

**Affiliations:** 1 Ophthalmology Department, Çerkezköy State Hospital, Tekirdağ, Turkey; 2 Ophthalmology Department, Cerrahpaşa Faculty of Medicine, Istanbul University – Cerrahpaşa, Istanbul, Turkey; 3 Ophthalmology Department, Ayancık State Hospital, Sinop, Turkey

**Keywords:** choroid hemorrhage, choroid neoplasms, choroidal neovascularization, foreign bodies, eye injuries

## Abstract

**Purpose:**

To report an occult intraocular foreign body mimicking choroidal melanoma.

**Methods:**

Medical records and imagings of the patient were retrospectively reviewed.

**Case description:**

A 76-year-old male was referred to our ocular oncology clinic with a suspicious hyperpigmented retinal lesion in the left eye. Biomicroscopy showed aphakia and peripheral iridectomy in the left eye. Fundoscopy revealed a pigmented, slightly elevated lesion on the macula of the left eye surrounded by diffuse atrophy. B-scan ultrasonography showed a preretinal hyperechoic lesion with posterior shadowing. There was no choroidal mass in B-scan or optical coherence tomography (OCT) imaging. On further questioning, it was disclosed that the patient had been hit by an iron fragment in the left eye forty years ago.

**Conclusion:**

Choroidal melanoma is a vision- and life-threatening intraocular malignant tumour. Various neoplastic, degenerative, and inflammatory conditions can simulate choroidal melanoma. A previous history of penetrating ocular trauma should lead the surgeon to re-evaluate a diagnosis of melanoma.

## Introduction

Choroidal melanoma is a vision- and life-threatening intraocular malignant tumour. Choroidal melanoma usually presents itself as a pigmented mass in fundoscopic examination. Treatment approaches include transpupillary thermotherapy, plaque radiotherapy, local resection, enucleation, or orbital exenteration [1]. Various neoplastic, degenerative and inflammatory conditions such as choroidal nevus, congenital hypertrophy of the retinal pigment epithelium, hemorrhagic retina pigment epithelium (RPE) detachment, massive subretinal hemorrhage due to neovascular age-related macular degeneration, choroidal hemangioma, peripheral exudative hemorrhagic chorioretinopathy, RPE hyperplasia, optic disc melanocytoma, solitary choroiditis, nodular posterior scleritis, and staphyloma can simulate choroidal melanoma [1], [2]. Due to the novel examination techniques and increased familiarity with confusing lesions, diagnosis has become more accurate in recent years. Among these pseudomelanomas, a retained ocular foreign body is a rare cause. We report a case of occult intraocular foreign body which mimics choroidal melanoma.

## Case description

**Figure 1:**
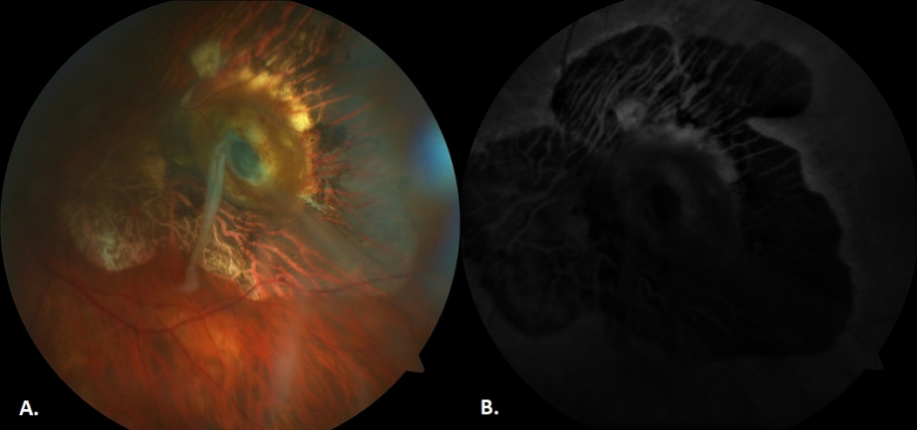
A. Fundus image shows pigmented and elevated lesion on the paramacular area, surrounded by diffuse atrophy. B. Fundus autofluorescence image shows a wide hypoautofluorescent area due to the RPE atrophy with regular borders and hyperautofluorescence along the margin of the lesion.

**Figure 2:**
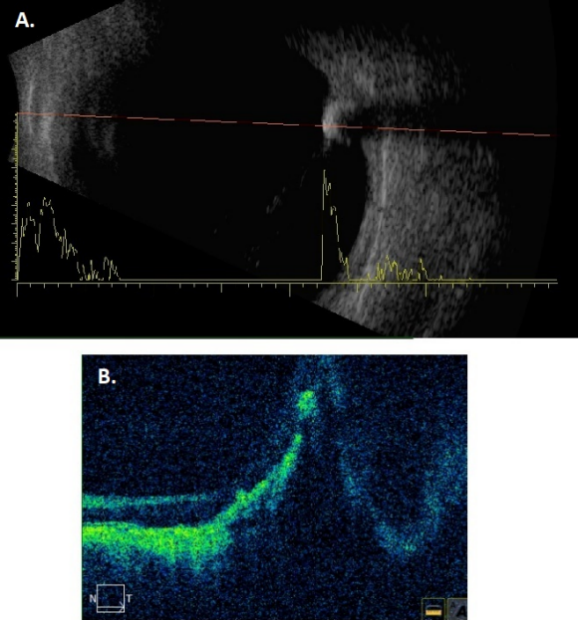
A. B-scan ultrasonography image shows a preretinal hyperechoic lesion with posterior shadowing. B. Optical coherence tomography image shows the preretinal localization of the foreign body with posterior shadowing. Both images do not disclose any choroidal mass.

A 76-year-old man with the suspicion of choroidal melanoma in his left eye was referred to our ocular oncology clinic. The best corrected visual acuity was 0.7 in the right eye, and hand movement from 30 cm in the left eye. On biomicroscopy cortical cataract in the right eye, and aphakia and peripheral iridectomy in the left eye were noted. Fundoscopy revealed posterior vitreous detachment in the right eye and a pigmented, slightly elevated lesion on the paramacular area of the left eye surrounded by diffuse atrophy (Figure 1A (Fig. 1)). Feeding vessel or retinal detachment was not observed. Grade 1 hypertensive retinopathy was present in both eyes. Fundus autofluorescence showed a wide hypoautofluorescent area due to the diffuse RPE atrophy with regular borders and hyperautofluorescence along the margin of the pigmented lesion (Figure 1B (Fig. 1)). B-scan ultrasonography disclosed a preretinal hyperechoic lesion with posterior shadowing (Figure 2A (Fig. 2)). Optical coherence tomography (OCT) showed the preretinal localization of the foreign body with posterior shadowing (Figure 2B (Fig. 2)). Neither USG nor OCT showed any choroidal mass (Figure 2 (Fig. 2)). When questioned in detail, it was revealed that the patient had been hit by an iron fragment in the left eye forty years ago.

## Discussion

Pseudomelanoma management can be challenging, and misdiagnosis may lead to severe therapeutic consequences. Cases of misdiagnosis which led to enucleation are reported in the literature [3], [4]. Different studies report various frequencies for pseudomelanoma diagnosis, possibly due to variable primary ophthalmic care conditions. In a retrospective analysis of 12,000 patients referred for choroidal melanoma, the frequency of pseudomelanoma was reported as 14.4% by Shields et al. [2]. Another study revealed a pseudomelanoma frequency of 53.7% (246/458) among eyes examined for choroidal melanoma [5].

The most common conditions that mimick choroidal melanoma are choroidal nevus, peripheral exudative hemorrhagic chorioretinopathy, and congenital hypertrophy of retina pigment epithelium (RPE), respectively [1]. Other conditions include hemorrhagic RPE detachment, massive subretinal hemorrhage due to neovascular age-related macular degeneration, choroidal hemangioma, RPE hyperplasia, choroidal metastasis, vasoproliferative tumors, and intraocular foreign body. The frequency of intraocular foreign body among the reasons of pseudomelanoma diagnosis was only 0.17% [2]. Chorioretinal tissue responds to injury by penetration with granulation, scar formation, and proliferation of retinal pigment epithelium [6], and therefore the lesion may be mistaken for melanoma.

In the presented case, the patient had a slightly elevated hyperpigmented lesion surrounded by RPE atrophy. No distinguishable foreign body was observed. The fundoscopic image resembled a melanoma, but USG and OCT scans revealed a high reflective foreign body. If the lesion was choroidal melanoma, we would expect low internal reflectivity (acoustic hollowness) on echography. Transillumination had no impact on differential diagnosis because of the posterior location of the lesion. Fluorescein angiography could not be obtained because the patient had chronic renal failure. The role of different diagnostic techniques in the differential diagnosis of melanoma can be seen in Table 1 (Tab. 1).

## Conclusion

Multimodal imaging can be helpful to make the correct diagnosis. Accurate diagnosis of choroidal melanoma is of utmost importance since the therapeutic choices can have serious side effects, including devastating operations. As projected, due to the novel diagnostic techniques and increased knowledge of mimicking lesions the incidence of misdiagnosis decreased. Antecedent history of penetrating ocular trauma should prompt the surgeon to re-evaluate a diagnosis of melanoma.

## Notes

### Authors’ ORCIDs

- Aslıhan Yılmaz Çebi: 0000-0001-7919-9099
- Bilge Batu Oto: 0000-0002-9729-2577
- Oğuzhan Kılıçarslan: 0000-0003-4061-2047
- Ahmet Murat Sarıcı: 0000-0002-9061-4385

### Ethics statement

This study was performed in accordance with the Declaration of Helsinki. Written informed consent was obtained from the patient.

### Competing interests

The authors declare that they have no competing interests.

## Figures and Tables

**Table 1:**
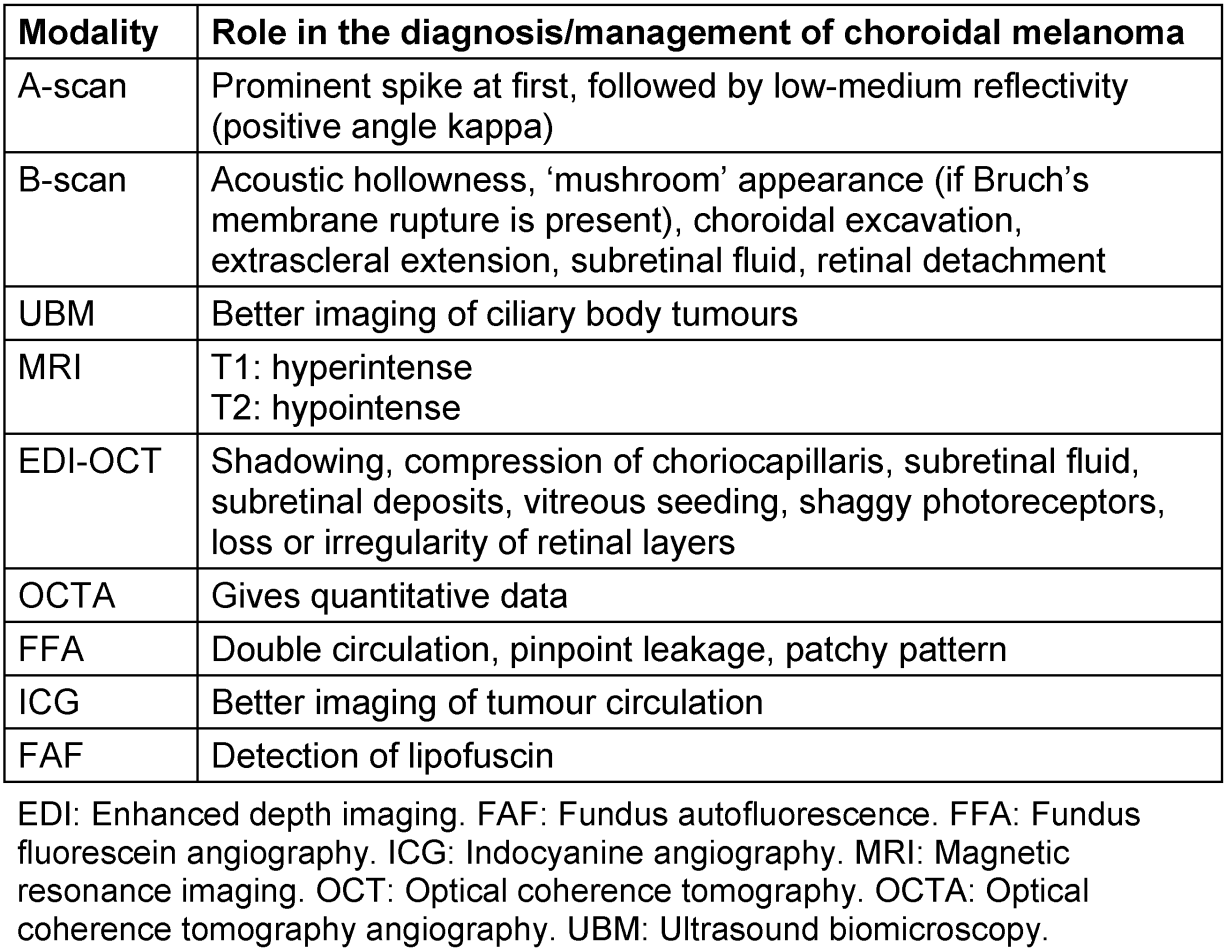
Different diagnostic techniques and their role in the diagnosis and/or management of choroidal melanoma
